# Caspase-6 does not contribute to the proteolysis of mutant huntingtin in the HdhQ150 knock-in mouse model of Huntington’s disease

**DOI:** 10.1371/4fd085bfc9973

**Published:** 2012-07-16

**Authors:** Christian Landles, Andreas Weiss, Sophie Franklin, David Howland, Gill Bates

## ABSTRACT

Huntington’s disease (HD) is a late-onset progressive neurodegenerative disorder characterised by irrepressible motor dysfunction, cognitive decline and psychiatric disturbances for which there is no effective disease-modifying treatment. The proteolytic cleavage of huntingtin (HTT) to generate N-terminal fragments has been proposed to be a key aspect of HD pathogenesis. In particular, it has been shown that HTT can be cleaved at amino acid 586 by caspase-6 (CASP6) and that prevention of cleavage at this site is neuroprotective and can rescue HD-related phenotypes in YAC transgenic HD mouse models. To determine the role that CASP6 plays in HTT proteolysis, we evaluated the effects of the genetic ablation of *Casp6* in the *Hdh*Q150 knock-in mouse model of HD. Here we show that the loss of CASP6 had no effect on the proteolysis of HTT, and did not modify the pattern of N-terminal HTT fragments that are present in the brains of these animals. Furthermore, we show that CASP6 ablation does not influence the steady-state levels of soluble HTT in the brains of presymptomatic mice. Therefore, we conclude that CASP6 is not necessary for HTT proteolysis in the *Hdh*Q150 mouse model of HD, and that targeting CASP6 as a therapeutic strategy should be approached with caution in the context of this complex disease.

* Corresponding author: Gillian P. Bates, Department of Medical and Molecular Genetics, King’s College London School of Medicine, 8th Floor Tower Wing, Guy’s Hospital, London, SE1 9RT, United Kingdom. Phone: +44 20 7188 3722; Fax: +44 20 7188 2585; Email: gillian.bates@kcl.ac.uk

## INTRODUCTION

Huntington’s disease (HD) is an autosomal-dominant neurodegenerative disorder with onset in midlife that is characterised by irrepressible motor dysfunction, personality changes, and cognitive decline ultimately leading to death 15-20 years after disease onset 1. HD belongs to a family of neurodegenerative diseases caused by mutations in which the expansion of a CAG trinucleotide repeat results in an aberrantly long polyglutamine (polyQ) tract in the encoded protein. In HD, the CAG expansion occurs within exon 1 of the *HTT* gene and leads to an elongated polyQ tract at the N-terminus of the HD protein huntingtin (HTT), a protein of many diverse functions 2, 3. Individuals with (CAG)_35 _or less remain unaffected, whereas those with (CAG)_40 _and above will develop HD within a normal lifespan. The length of the CAG expansion is inversely correlated with age of disease onset, with repeats at the higher end of the spectrum causing the juvenile form of HD.

Numerous HD mouse models have been developed and are being used to study the pathogenic pathways involved in HD 1. The *Hdh*Q150 knock-in mouse carries ~150 CAG repeats which have been inserted into the mouse HD gene (*Hdh*) 4. It has been proposed that the accumulation of a critical concentration of N-terminal HTT fragments represents a rate-limiting step in the onset and progression of HD-related phenotypes in this model 5, 6. Numerous studies suggest that the smallest N-terminal HTT fragments are pivotal to the molecular pathogenesis of HD. Human post-mortem studies showed that only N-terminal antibodies detect nuclear inclusions in patient brains 7 and formic acid solubilisation released a small N-terminal fragment 8. In cell models, HTT has been shown to be cleaved into smaller fragments called cp-A and cp-B 8 or cp-1 and cp-2 9, 10. Furthermore, using the *Hdh*Q150 knock-in model we recently demonstrated that full-length HTT is cleaved into a number of N-terminal HTT fragments, the smallest of which is an exon 1 HTT protein 6 .

The ensuing search for sites of proteolytic cleavage of human HTT raised the hypothesis that the cleavage of HTT by proteases contributes to the pathogenesis of HD. Studies have identified active caspase-3 (HTT-513 and HTT-552) and caspase-6 (HTT-586) sites 11, calpain sites (HTT-469 and HTT-536) 12 , 13, and recently an MMP-10 site (HTT-402) 14. Additionally, a number of less well-defined HTT cleavage fragments have been isolated from HD post-mortem brains 8 and from a number of HD model systems, for which the precise cleavage sites and proteases involved remain elusive 9, 10, 15, 16, 17, 18, 19. The prevention of cleavage at the caspase-6 but not caspase-3 sites was found to be protective in the YAC128 HD mouse model 20. In that study, mutation of the aspartate at amino acid 586 ameliorated HD-phenotypes, implying that the caspase-6 mediated cleavage of HTT is critical in the pathogenesis of HD. The HTT-586 fragment could itself be pathogenic and / or cleavage at this site might initiate further proteolytic events that generate smaller N-terminal fragments with detrimental effects: the cascade hypothesis. Alternatively, the aspartate substitution may alter the conformation of HTT without altering cleavage, and it could be this structural change that improves the phenotype of these mice.

We previously mapped the location of the proteolytic cleavage sites required to generate the N-terminal HTT fragments that are present in the *Hdh*Q150 HD model, and this suggested that *Fragment 8* might be generated by caspase-6 6. To test this hypothesis, we crossed caspase-6 (*Casp6*)-deficient (*Casp6* ^–/–^) mice with the *Hdh*Q150 mouse model of HD and analysed the pattern of N-terminal HTT fragments. This genetic approach would test whether CASP6 actually cleaves HTT at amino acid 586* in vivo*, and also assess the so called cascade hypothesis should cleavage at HTT-586 be inhibited. In concurrence with our previous findings, we were able to identify the same 14 prominent N-terminal mutant HTT fragments in the brain of the *Hdh*Q150 mouse. Interestingly, we showed that the ablation of CASP6 had no effect on the production of *Fragment 8* or any other N-terminal HTT proteolytic fragments. In addition, the ablation of CASP6 had no effect on the levels of the full-length HTT protein. Therefore, CASP6 does not play a direct role in the molecular pathogenesis that occurs in *Hdh*Q150 mice through the modulation of HTT proteolysis or modifying the levels of mutant HTT.

## MATERIALS AND METHODS

**Mouse Breeding, Maintenance, Genotyping, and CAG Repeat Sizing.** All mouse experiments were performed under the Animals Scientific Procedures Act (1986) under project and personal licences approved and issued by the Home Office. The *Hdh*Q150 knock-in mice were maintained, genotyped, and CAG repeat-sized as previously described 21 . The mean repeat size was 179 ± 7.6 (±S.D.). The *Casp6* ^–/– ^mouse was created by Taconic Biosciences and genotyped as previously described 22 . The *Casp6* mice were maintained by backcrossing *Casp6* ^+/– ^males to C57BL/6J females (Charles River, A003). *Hdh* ^+/Q150^::*Casp6* ^–/– ^(Dble) and* Hdh* ^+/Q150^::*Casp6* ^+/+ ^(*Hdh* ^+/Q150^) mice were generated by crossing *Casp6* ^+/– ^males with C57BL/6J *Hdh* ^+/Q150^::*Casp6* ^+/– ^females. Mouse brains were snap-frozen in liquid nitrogen and stored at –80°C.

**Immunoprecipitation.** Whole brains were homogenised in ice-cold HEPES buffer (50 mM HEPES/NaOH (pH7.0), 150 mM NaCl, 10 mM EDTA, 1.0% Nonidet P-40, 0.5% sodium deoxycholate, 0.1% SDS) with 10 mM DTT, 1 mM PMSF, and complete protease inhibitor mixture (Roche). Lysates were processed for western blotting or immunoprecipitation as described 6. For immunoprecipitation, either 2 µg mIgG, 2B7, S830, or 3B5H10 was used. (See Table 1 and 2 for antibody details).

**Western Immunoblotting.** For western blotting, 30 µg of total protein or 6 µl of immunoprecipitate in Laemmli buffer were denatured at 75°C for 10 min and separated by 8% or 10% SDS-PAGE, blotted onto nitrocellulose membranes (Whatman) and immunoprobed as described 6. Primary antibodies: MW1 (1:1,000, 23); CASP6 (1:125); β-ACTIN (1:10,000). Secondary antibodies: anti-mouse HRP (1:3,000); anti-rabbit HRP (1:3,000). (See Table 1 and 2 for antibody details).

**TR-FRET.** Time-resolved Förster resonance energy transfer (TR-FRET) was performed as described previously 24. 2B7 antibody was labelled with terbium cryptate donor fluorophore (CisBio). MW1 was labelled with D2 acceptor fluorophore (CisBio). MAB2166 was labelled with Alexa-fluor (A)488 (Invitrogen).

**Table 1: Primary Antibodies:** 

Antibody	Epitope	Description	Source
mIgG	–	Mouse IgG	Alpha Diagnostic (#20008-250)
2B7	HTT aa1-17	Monoclonal	25
S830	Exon1 (53Q)	Polyclonal	26
3B5H10	N171 (65Q)	Monoclonal	Sigma-Aldrich (P1874)
MW1	PolyQ	Monoclonal	23
MAB2166	HTT aa443-457	Monoclonal	Millipore (MAB2166)
CASP6	CASP6 cleavage site	Polyclonal	Cell Signalling (#9762)
Beta-ACTIN	ACTIN aa1-15	Monoclonal	Abcam (ab6276)

**Table 2: Secondary Antibodies:** 

Antibody	Description	Source
Anti-mouse HRP	Polyclonal-rabbit	DAKO (P0260)
Anti-rabbit HRP	Polyclonal-swine	DAKO (P0449)

## RESULTS

***Casp6^–/– ^*mice show no CASP6 expression in the brain.** Exons 2-5 of the *Casp6* gene, which encodes the catalytic domain of the caspase-6 (CASP6) protein, were removed to generate *Casp6* ^–/–^ mice. *Casp6* ^–/– ^mice are viable and fertile, and do not show any overt phenotypes 22. We began by performing western blots to ensure that these mice do not express CASP6 and to verify the genotypes. Protein lysates were prepared from half brains of 2 month old wild type and *Casp6* ^–/– ^mice and analysed by western blotting using an anti-CASP6 antibody. As previously reported 22, we found that the *Casp6* ^–/– ^mice constitutively lacked CASP6 as compared to wild type littermates (Fig. 1).

**Figure 1. CASP6 expression in *Casp6*^–/–^ and wild type brains**

**Figure:**
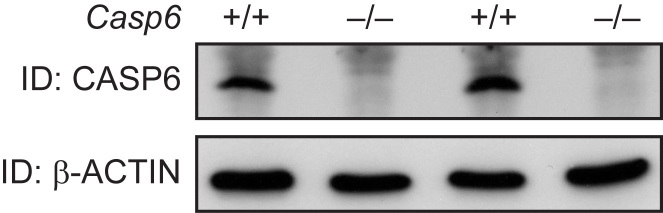
Western blotting of total brain lysates from 2 month old mice using an anti-CASP6 antibody shows that CASP6 was absent in *Casp6* ^–/– ^samples when compared to wild type (*Casp6* ^+/+^) samples. Beta-ACTIN served as a loading control.* ID* = Immunodetection.

**Genetic ablation of *Casp6* does not modify HTT proteolysis in *Hdh*Q150 knock-in mice.** To identify N-terminal HTT fragments in the brains of *Hdh*Q150 knock-in mice, we previously used a combination of immunoprecipitation and western blotting that identified 14 prominent N-terminal fragments, which in addition to the full-length protein, could be readily detected in the cytoplasmic but not nuclear fractions. These fragments were present at all ages and did not arise as a consequence of the pathogenic process. Furthermore, in an attempt to determine the source of some of the N-terminal HTT fragments, proteolytic digests were performed with caspase enzymes which indicated that *Fragment 8* may terminate at the caspase-6 HTT-586 cleavage site 6. In order to corroborate this observation, we investigated whether the presence of this proteolytic fragment, along with all other observed N-terminal HTT fragments, was modulated upon the genetic ablation of CASP6.* Casp6* ^+/– ^males were bred to *Hdh* ^+/Q150^::*Casp6* ^+/– ^females to generate *Hdh* ^+/Q150 ^mice on either a *Casp6* wild type *Hdh* ^+/Q150^::*Casp6* ^+/+ ^(*Hdh* ^+/Q150^) or *Casp6* knock-out *Hdh* ^+/Q150^::*Casp6* ^–/– ^(Dble) background. We obtained at least 12 mice for each genotype and all groups were well matched for their CAG repeat size (*Hdh* ^+/Q150 ^179 ±7.5 (±S.D); (Dble 179 ±9.5 (±S.D)).

To compare the pattern of HTT proteolytic fragments, we applied a combination of HTT immunoprecipitation and immunodetection from whole brain lysates prepared from *Hdh* ^+/Q150^ and Dble mice aged 2 months (Fig. 2). Mutant HTT was immunoprecipitated with antibodies that recognize epitopes at the N-terminus (2B7; S830; or 3B5H10) (Fig. 2B-C) and immunodetected with MW1, an antibody that recognizes the expanded polyQ tract and, therefore, does not detect wild type HTT. A mouse IgG (mIgG) antibody was used as a negative control (Fig. 2A), and *Casp6* genotypes were confirmed by western blotting (Fig. 2E). In both *Hdh* ^+/Q150^ and Dble samples we were able to identify all of the N-terminal fragments identified previously, including *Fragment 8* (the proposed caspase-6 HTT-586 fragment) 6. Therefore, the proteolysis of full-length HTT, to generate *Fragment 8* or any other N-terminal HTT fragments was unaltered in the absence of CASP6.

**Figure 2. Analysis of N-terminal HTT fragments in *Hdh*^+/Q150^ and Dble mouse brains**

**Figure:**
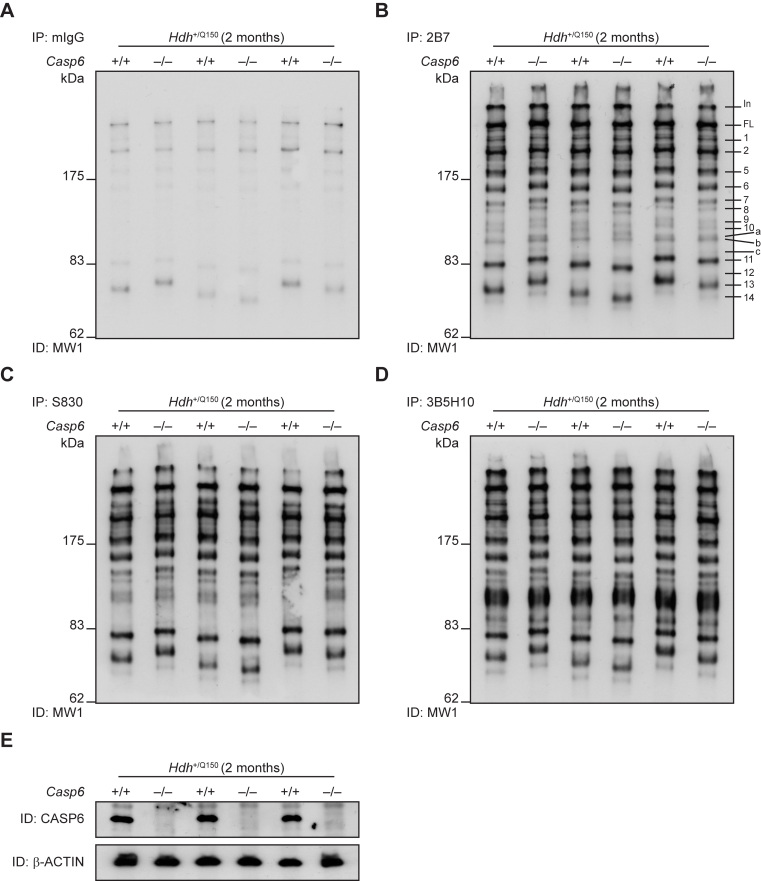
Immunoprecipitation of mutant HTT from *Hdh* ^+/Q150^ or Dble samples at 2 months of age with either: (**A**) mIgG (control), (**B**) 2B7, (**C**) S830, or (**D**) 3B5H10, and immunodetection with MW1. Fourteen prominent N-terminal fragments can be detected (*Fragments 1-14*), in addition to 3 weaker fragments (*Fragments a-c*), numbered and labelled in panel (B). (**E**) *Casp6* genotypes were confirmed by western blotting, Beta-ACTIN served as a loading control. *IP* = Immunoprecipitation; *ID* = Immunodetection; *In* = Interface between stacking and resolving gel; *FL* = full-length protein. (*n* = 3/genotype).

**Steady state wild type and mutant HTT levels are not changed in the brains of presymptomatic *Hdh^+^*^/Q150^ and Dble mice.** To quantify the relative levels of mutant HTT with respect to total HTT, brain lysates from 2 month-old mice were subjected to the highly sensitive TR-FRET assay (Fig. 3). This assay uses the labelled antibody pair: 2B7-Terbium cryptate and MAB2166-Alexa488 to quantify levels of total HTT, or 2B7-Terbium cryptate and MW1-D2 to quantify levels of mutant HTT (the wild type mouse protein is not detected by MW1) 24. There were no significant differences in the steady state levels of either mutant HTT (Fig. 3A), or total HTT (Fig. 3B) between *Hdh* ^+/Q150 ^and Dble samples, when wild type (*Hdh* ^+/+^::*Casp6* ^+/+^) fractions served as controls. Mutant HTT could not be detected in wild type fractions as expected (Fig. 3A), however, the 2B7-MAB2166 signal was lower in the *Hdh* ^+/Q150 ^and Dble fractions than in wild type, largely because the 2B7-MAB2166 FRET is weaker in the presence of highly expanded CAG-repeats present in the mutant HTT protein (Fig. 3B). Therefore these data show that the ablation of CASP6 does not influence soluble levels of wild type or mutant HTT in the *Hdh*Q150 brain.

**Figure 3. Steady-state levels of mutant HTT and total HTT are unchanged between *Hdh*^+/Q150^ and Dble samples**

**Figure:**
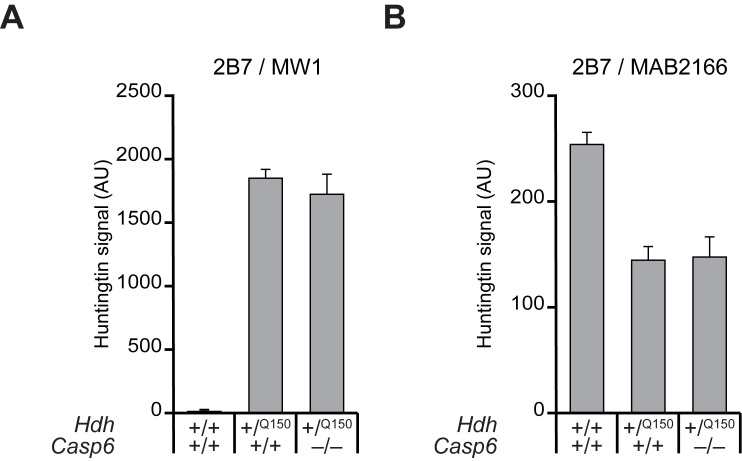
TR-FRET was performed on total brain lysates prepared from *Hdh* ^+/Q150 ^or Dble samples at 2 months of age. (**A**) 2B7-MW1, to quantify levels of mutant HTT, (**B**) 2B7-MAB2166, to quantify levels of total HTT. Wild type fractions served as controls. The 2B7-MAB2166 signal was higher in wild type lysates than the *Hdh* ^+/Q150 ^or Dble lysates largely because the TR-FRET signal is reduced in the presence of a long polyQ tract. (*n* = 4/genotype).

## DISCUSSION

Numerous studies provided evidence to show that HTT can be cleaved by proteases, however, the relevance of this finding to the pathogenesis of HD remains elusive. Specifically, there is evidence to indicate that protecting the CASP6 cleavage of HTT at amino acid 586 is neuroprotective and can rescue HD-related phenotypes 20. To extend this analysis and test this hypothesis *in vivo*, we investigated the effects of the genetic ablation of *Casp6* in the *Hdh*Q150 mouse model of HD. In this study, we showed that *Casp6* ^–/–^ mice do not express the CASP6 protein. We crossed the *Hdh*Q150 mice to *Casp6* mice and employed our previously described unbiased immunoprecipitation and immunodetection strategy to detect N-terminal HTT proteolytic cleavage fragments in the brains of *Hdh* ^+/Q150 ^mice that did or did not express CASP6. In accordance with our previous findings, we could detect the C-terminus of 14 prominent mutant HTT N-terminal fragments (*Fragments 1-14*) and three additional fragments (*Fragments a-c*) in the brain at 2 months of age 6. We found that the ablation of CASP6 did not modify the production of *Fragment 8* (the previously proposed caspase-6 HTT-586 fragment), or any other N-terminal HTT fragment. Since the ablation of CASP6 had no effect on the proteolysis of mutant HTT we were unable to test the cascade hypothesis to determine whether the production of one fragment is essential for the generation of others.

Given that *Fragment 8 *is still present in the absence of CASP6, it must therefore be generated by an alternative protease that cleaves either at the HTT-586 site or at a different position nearby. If the cleavage occurs at HTT-586, an alternative protease also recognises the CASP6 recognition motif and therefore, redundancy would be expected to ensure that the site is cleaved irrespective of whether CASP6 is present or absent. This could account for a compensatory mechanism in these constitutive knock-out mice. It would also confound therapeutic approaches directed at CASP6 inhibition. In concurrence with our findings, it has recently been demonstrated that the ablation of CASP6 activity in an aged BACHD mouse model did not show a reduction in the proteolysis of HTT at amino acid 586 27, and our work now extends this observation to two different HD mouse models.

The ablation of CASP6 in BACHD mice aged 13 months resulted in a reduction of both wild type and mutant HTT levels, possibly due to the activation of protein clearance pathways 27. We performed TR-FRET to quantify levels of both mutant HTT and total HTT and found that the absence of CASP6 had no effect on soluble HTT levels in presymptomatic mice. Such a difference in the effect of ablating CASP6 on HTT levels in young (2 month) *Hdh*Q150 and aged (13 month) BACHD mice is not clear. It could be that protein clearance pathways are regulated by CASP6 only at later stages of disease, particularly when toxic HTT aggregates are present.

Preventing the formation of N-terminal HTT fragments is an attractive therapeutic strategy that needs to be understood. Our data suggest that CASP6 is not necessary for the proteolysis of mutant HTT in *Hdh*Q150 mice and by implication does not play a direct role in HD pathogenesis in this mouse model via this mechanism. Our work highlights the importance of identifying the source of HTT proteolytic fragments to allow the design of rational therapeutic strategies when considering how one should combat the many HD-related phenotypes associated with this complex neurodegenerative disease.

## COMPETING INTERESTS

AW is an employee of Novartis AG.

## ACKNOWLEDGEMENTS

We thank Dr. Anna Bobrowska and members of the Neurogenetics Laboratory for critical advice on the manuscript.

## References

[1] Bates GP, Benn C (2002) The Polyglutamine Diseases. In: Bates GP, Harper PS, Jones AL, editors. Huntington's Disease. 3rd ed. Oxford: Oxford University Press. pp. 429-472.

[2] Landles C, Bates GP (2004) Huntingtin and the molecular pathogenesis of Huntington's disease. Fourth in molecular medicine review series. EMBO Rep 5: 958-963. 10.1038/sj.embor.7400250 PMC1299150 15459747

[3] Harjes P, Wanker EE (2003) The hunt for huntingtin function: interaction partners tell many different stories. Trends Biochem Sci 28: 425-433. 10.1016/S0968-0004(03)00168-3 12932731

[4] Lin CH, Tallaksen-Greene S, Chien WM, Cearley JA, Jackson WS, et al. (2001) Neurological abnormalities in a knock-in mouse model of Huntington's disease. Hum Mol Genet 10: 137-144. 10.1093/hmg/10.2.137 11152661

[5] Woodman B, Butler R, Landles C, Lupton MK, Tse J, et al. (2007) The Hdh(Q150/Q150) knock-in mouse model of HD and the R6/2 exon 1 model develop comparable and widespread molecular phenotypes. Brain Res Bull 72: 83-97. 10.1016/j.brainresbull.2006.11.004 17352931

[6] Landles C, Sathasivam K, Weiss A, Woodman B, Moffitt H, et al. (2010) Proteolysis of mutant huntingtin produces an exon 1 fragment that accumulates as an aggregated protein in neuronal nuclei in Huntington disease. J Biol Chem 285: 8808-8823. 10.1074/jbc.M109.075028 PMC2838303 20086007

[7] DiFiglia M, Sapp E, Chase KO, Davies SW, Bates GP, et al. (1997) Aggregation of huntingtin in neuronal intranuclear inclusions and dystrophic neurites in brain. Science 277: 1990-1993. 10.1126/science.277.5334.1990 9302293

[8] Lunkes A, Lindenberg KS, Ben-Haiem L, Weber C, Devys D, et al. (2002) Proteases acting on mutant huntingtin generate cleaved products that differentially build up cytoplasmic and nuclear inclusions. Mol Cell 10: 259-269. 10.1016/s1097-2765(02)00602-0 12191472

[9] Ratovitski T, Nakamura M, D'Ambola J, Chighladze E, Liang Y, et al. (2007) N-terminal proteolysis of full-length mutant huntingtin in an inducible PC12 cell model of Huntington's disease. Cell Cycle 6: 2970-2981. 10.4161/cc.6.23.4992 18156806

[10] Ratovitski T, Gucek M, Jiang H, Chighladze E, Waldron E, et al. (2009) Mutant huntingtin N-terminal fragments of specific size mediate aggregation and toxicity in neuronal cells. J Biol Chem 284: 10855-10867. 10.1074/jbc.M804813200 PMC2667772 19204007

[11] Wellington CL, Ellerby LM, Gutekunst CA, Rogers D, Warby S, et al. (2002) Caspase cleavage of mutant huntingtin precedes neurodegeneration in Huntington's disease. J Neurosci 22: 7862-7872. 10.1523/JNEUROSCI.22-18-07862.2002 PMC6758089 12223539

[12] Kim YJ, Yi Y, Sapp E, Wang Y, Cuiffo B, et al. (2001) Caspase 3-cleaved N-terminal fragments of wild-type and mutant huntingtin are present in normal and Huntington's disease brains, associate with membranes, and undergo calpain-dependent proteolysis. Proc Natl Acad Sci U S A 98: 12784-12789. 10.1073/pnas.221451398 PMC60131 11675509

[13] Gafni J, Hermel E, Young JE, Wellington CL, Hayden MR, et al. (2004) Inhibition of calpain cleavage of huntingtin reduces toxicity: accumulation of calpain/caspase fragments in the nucleus. J Biol Chem 279: 20211-20220. 10.1074/jbc.M401267200 14981075

[14] Miller JP, Holcomb J, Al-Ramahi I, de Haro M, Gafni J, et al. (2010) Matrix metalloproteinases are modifiers of huntingtin proteolysis and toxicity in Huntington's disease. Neuron 67: 199-212. 10.1016/j.neuron.2010.06.021 PMC3098887 20670829

[15] Kim YJ, Sapp E, Cuiffo BG, Sobin L, Yoder J, et al. (2006) Lysosomal proteases are involved in generation of N-terminal huntingtin fragments. Neurobiol Dis 22: 346-356. 10.1016/j.nbd.2005.11.012 16423528

[16] Schilling G, Klevytska A, Tebbenkamp AT, Juenemann K, Cooper J, et al. (2007) Characterization of huntingtin pathologic fragments in human Huntington disease, transgenic mice, and cell models. J Neuropathol Exp Neurol 66: 313-320. 10.1097/nen.0b013e318040b2c8 17413322

[17] Sun B, Fan W, Balciunas A, Cooper JK, Bitan G, et al. (2002) Polyglutamine repeat length-dependent proteolysis of huntingtin. Neurobiol Dis 11: 111-122. 10.1006/nbdi.2002.0539 12460551

[18] Tanaka Y, Igarashi S, Nakamura M, Gafni J, Torcassi C, et al. (2006) Progressive phenotype and nuclear accumulation of an amino-terminal cleavage fragment in a transgenic mouse model with inducible expression of full-length mutant huntingtin. Neurobiol Dis 21: 381-391. 10.1016/j.nbd.2005.07.014 16150600

[19] Waldron-Roby E, Ratovitski T, Wang X, Jiang M, Watkin E, et al. (2012) Transgenic mouse model expressing the caspase 6 fragment of mutant huntingtin. J Neurosci 32: 183-193. 10.1523/JNEUROSCI.1305-11.2012 PMC3306223 22219281

[20] Graham RK, Deng Y, Slow EJ, Haigh B, Bissada N, et al. (2006) Cleavage at the caspase-6 site is required for neuronal dysfunction and degeneration due to mutant huntingtin. Cell 125: 1179-1191. 10.1016/j.cell.2006.04.026 16777606

[21] Sathasivam K, Lane A, Legleiter J, Warley A, Woodman B, et al. (2010) Identical oligomeric and fibrillar structures captured from the brains of R6/2 and knock-in mouse models of Huntington's disease. Hum Mol Genet 19: 65-78. 10.1093/hmg/ddp467 PMC2792149 19825844

[22] Uribe V, Wong BK, Graham RK, Cusack CL, Skotte NH, et al. (2012) Rescue from excitotoxicity and axonal degeneration accompanied by age-dependent behavioral and neuroanatomical alterations in caspase-6-deficient mice. Hum Mol Genet 21: 1954-1967. 10.1093/hmg/dds005 PMC3315204 22262731

[23] Ko J, Ou S, Patterson PH (2001) New anti-huntingtin monoclonal antibodies: implications for huntingtin conformation and its binding proteins. Brain Res Bull 56: 319-329. 10.1016/s0361-9230(01)00599-8 11719267

[24] Weiss A, Abramowski D, Bibel M, Bodner R, Chopra V, et al. (2009) Single-step detection of mutant huntingtin in animal and human tissues: a bioassay for Huntington's disease. Anal Biochem 395: 8-15. 10.1016/j.ab.2009.08.001 19664996

[25] Weiss A, Grueninger S, Abramowski D, Giorgio FP, Lopatin MM, et al. (2011) Microtiter plate quantification of mutant and wild-type huntingtin normalized to cell count. Anal Biochem 410: 304-306. 10.1016/j.ab.2010.11.044 21134349

[26] Sathasivam K, Woodman B, Mahal A, Bertaux F, Wanker EE, et al. (2001) Centrosome disorganization in fibroblast cultures derived from R6/2 Huntington's disease (HD) transgenic mice and HD patients. Hum Mol Genet 10: 2425-2435. 10.1093/hmg/10.21.2425 11689489

[27] Gafni J, Papanikolaou T, Degiacomo F, Holcomb J, Chen S, et al. (2012) Caspase-6 Activity in a BACHD Mouse Modulates Steady-State Levels of Mutant Huntingtin Protein But Is Not Necessary for Production of a 586 Amino Acid Proteolytic Fragment. J Neurosci 32: 7454-7465. 10.1523/JNEUROSCI.6379-11.2012 PMC3454486 22649225

